# Molecular cytogenetic characterization of wheat–*Elymus repens* chromosomal translocation lines with resistance to Fusarium head blight and stripe rust

**DOI:** 10.1186/s12870-019-2208-x

**Published:** 2019-12-27

**Authors:** Biran Gong, Wei Zhu, Sanyue Li, Yuqi Wang, Lili Xu, Yi Wang, Jian Zeng, Xing Fan, Lina Sha, Haiqin Zhang, Pengfei Qi, Lin Huang, Guoyue Chen, Yonghong Zhou, Houyang Kang

**Affiliations:** 1 State Key Laboratory of Crop Gene Exploration and Utilization in Southwest China, Sichuan Agricultural University, Chengdu, 611130 Sichuan China; 2 Triticeae Research Institute, Sichuan Agricultural University, Chengdu, 611130 Sichuan China; 3 College of Resources, Sichuan Agricultural University, Chengdu, 611130 Sichuan China

**Keywords:** Chromosomal translocation line, *Elymus repens*, Fusarium head blight (FHB), Stripe rust

## Abstract

**Background:**

Fusarium head blight (FHB) caused by the fungus *Fusarium graminearum* Schwabe and stripe rust caused by *Puccinia striiformis* f. sp. *tritici* are devastating diseases that affect wheat production worldwide. The use of disease-resistant genes and cultivars is the most effective means of reducing fungicide applications to combat these diseases. *Elymus repens* (2*n* = 6*x* = 42, StStStStHH) is a potentially useful germplasm of FHB and stripe rust resistance for wheat improvement.

**Results:**

Here, we report the development and characterization of two wheat*–E. repens* lines derived from the progeny of common wheat–*E. repens* hybrids. Cytological studies indicated that the mean chromosome configuration of K15–1192-2 and K15–1194-2 at meiosis were 2*n* = 42 = 0.86 I + 17.46 II (ring) + 3.11 II (rod) and 2*n* = 42 = 2.45 I + 14.17 II (ring) + 5.50 II (rod) + 0.07 III, respectively. Genomic and fluorescence in situ hybridization karyotyping and simple sequence repeats markers revealed that K15–1192-2 was a wheat–*E. repens* 3D/?St double terminal chromosomal translocation line. Line K15–1194-2 was identified as harboring a pair of 7DS/?StL Robertsonian translocations and one 3D/?St double terminal translocational chromosome. Further analyses using specific expressed sequence tag-SSR markers confirmed that the wheat–*E. repens* translocations involved the 3St chromatin in both lines. Furthermore, compared with the wheat parent Chuannong16, K15–1192-2 and K15–1194-2 expressed high levels of resistance to FHB and stripe rust pathogens prevalent in China.

**Conclusions:**

Thus, this study has determined that the chromosome 3St of *E. repens* harbors gene(s) highly resistant to FHB and stripe rust, and chromatin of 3St introgressed into wheat chromosomes completely presented the resistance, indicating the feasibility of using these translocation lines as novel material for breeding resistant wheat cultivars and alien gene mining.

## Background

Fusarium head blight (FHB), mainly caused by the ascomycete fungus *Fusarium graminearum* Schwabe [telomorph, *Gibberella zeae* (Schw.) Petch], is an important disease of wheat (*Triticum aestivum* L.) worldwide [1, 2]. FHB causes significant yield losses, as well as reduced grain quality and functionality, owing to Fusarium-damaged kernels and mycotoxin contamination (mainly deoxynivalenol), which threaten food and feed security [3, 4]. Breeding resistant cultivars is generally considered the most effective and environmentally friendly strategy to control FHB [5]. To date, more than 100 unique quantitative trait loci (QTLs) have been reported on the 21 chromosomes of 50 wheat sources of resistant cultivars [6, 7]. Additionally, only a few formally designated FHB-resistance genes originated from the wild relatives of wheat, such as *Fhb3* derived from *Leymus racemosus*, *Fhb6* derived from *Elymus tsukushiensis*, and *Fhb7* derived from *Thinopyrum ponticum* [8–10]. Therefore, the discovery, development, and characterization of more new resistance sources will provide breeders with a wider choice of germplasm [7, 10].

Stripe rust, caused by *Puccinia striiformis* Westend. f. sp. *tritici* Eriks. (*Pst*), is a devastating disease to wheat production in many regions of the world [11]. New disease-resistant genes and cultivars are the most effective means of reducing the amounts of fungicides applied to combat this disease [12]. At present, 81 formally designated and 67 provisionally designated stripe rust resistance (*Yr*) genes and more than 330 QTLs are known to be distributed in common wheat and its relatives [13, 14]. However, most of the resistance genes (such as *Yr1–4*, *Yr6–10*, *Yr17*, *Yr20–22*, *Yr24–29*, and *Yr43*) are ineffective against the newly emerged virulent stripe rust race V26/Gui22 and its variants [15, 16]. Wild relatives of common wheat contain a large number of genes conferring desirable traits that can be exploited for wheat improvement [17]. For example, 23 formally designated genes are derived from wild wheat-related species in the tertiary gene pool, including *Secale cereale*, *Dasypyrum villosum*, *Thinopyrum intermedium*, *Th. ponticum*, and several *Aegilops* species [13, 18]. Hence, the identification of new resistance sources in adapted germplasm is an important and long-term objective in achieving durable and broad-spectrum resistance.

As an important wild relative of wheat, *Elymus repens* (L.) Gould [syn. *Agropyron repens* (L.) P. Beauvoir, *Elytrigia repens* (L.) Deskv. Ex. Nevski, and *Triticum repens* L.] possesses the StStStStHH genome and is distributed widely throughout the world [19]. It is a valuable species for wheat improvement because it tolerates a variety of soil types, heavy metals, and cold stress [20]. To date, there are few reports regarding its disease resistance. Zeng et al. [21] developed eight wheat–*E. repens* introgression lines with chromosomal numbers ranging from 42 to 56. These lines are resistant to FHB compared with the control cultivars *T. aestivum* “Roblin” and “Crocus”. It was concluded that these lines carry the FHB-resistance gene from *E. repens*. To transfer desirable traits from *E. repens* into wheat cultivars of the Sichuan Basin, China, we produced many progeny lines by crossing and backcrossing the wheat–*E. repens* line P1142-1-2 (2*n* = 56) with the native wheat cultivars. The present study was undertaken to develop and characterize wheat–*E. repens* translocation lines using genomic in situ hybridization (GISH), fluorescence in situ hybridization (FISH), and molecular markers. Additionally, their effects on FHB and stripe rust resistance and agronomical traits were evaluated.

## Results

### Meiotic behavior of the derivative lines

**Fig. 1:**
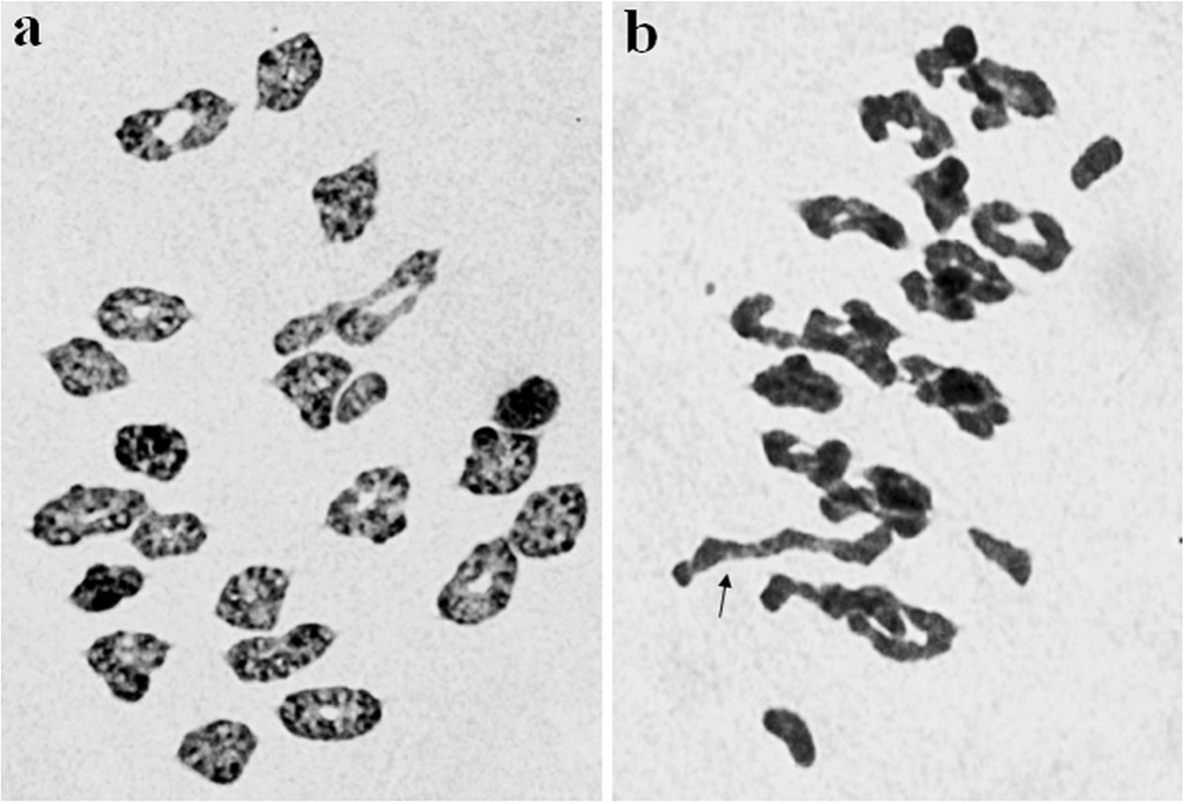
Meiotic metaphase I pairing analysis of K15–1192-2 (**a**) and K15–1194-2 (**b**). a, 2*n* = 42 = 2 I + 20 II (ring); b, 2*n* = 42 = 3 I + 16 II (ring) + 2 II (rod) + 1 III. The arrow indicate the trivalent

Chromosomal pairing at meiotic metaphase I in PMCs of K15–1192-2 was high, with an average chromosomal configuration of 0.86 univalents + 17.46 ring bivalents + 3.11 rod bivalents scored in ~ 50 PMCs per plant (Fig. 1a). Line K15–1194-2 had an average meiotic configuration of 2*n* = 42 = 2.45 I + 14.17 II (ring) + 5.50 II (rod) + 0.07 III per PMC (Fig. 1b). No lagging chromosomes or bridges were observed at anaphase I and II.

### GISH and FISH analyses

**Fig. 2:**
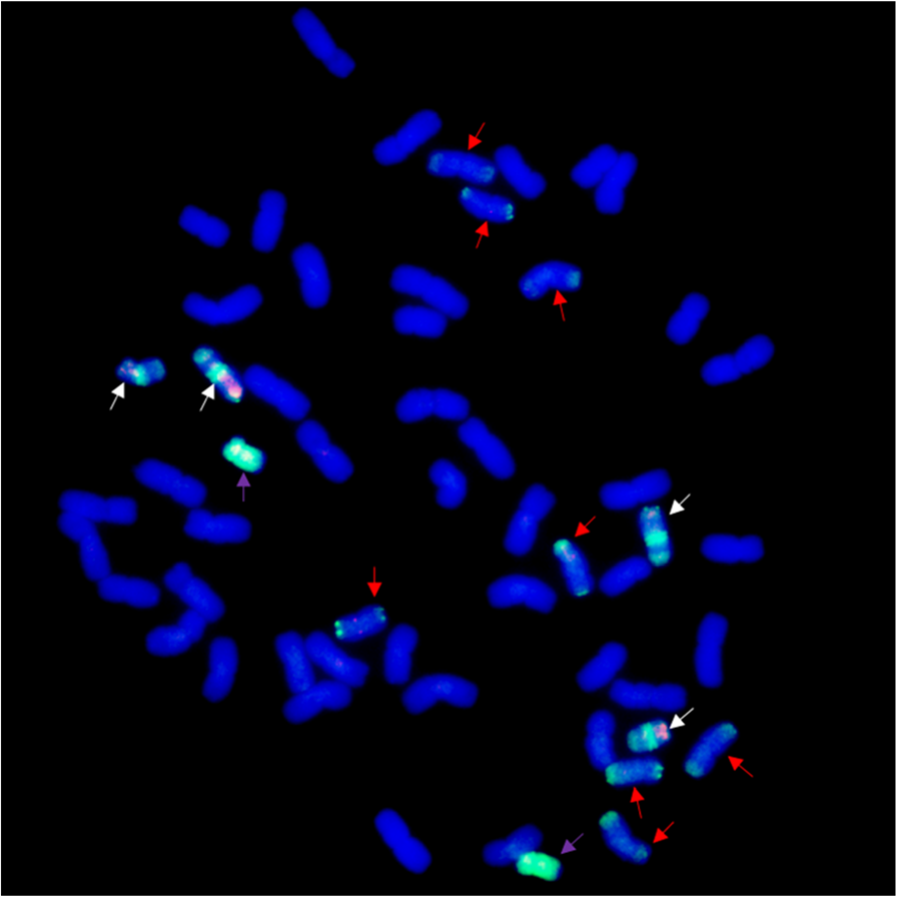
The genomic constitution of P1142-1-2 as revealed by multicolor GISH analysis. The probes used for in situ hybridization were *Pse. strigosa* DNA (green) and *H. bogdanii* DNA (red), which containing two St-genome chromosomes (purple arrows), four chromosomes with wheat, H, and St genome large fragments (white arrows), plus eight wheat chromosomes with double St-genome terminal translocations (red arrows)

**Fig. 3:**
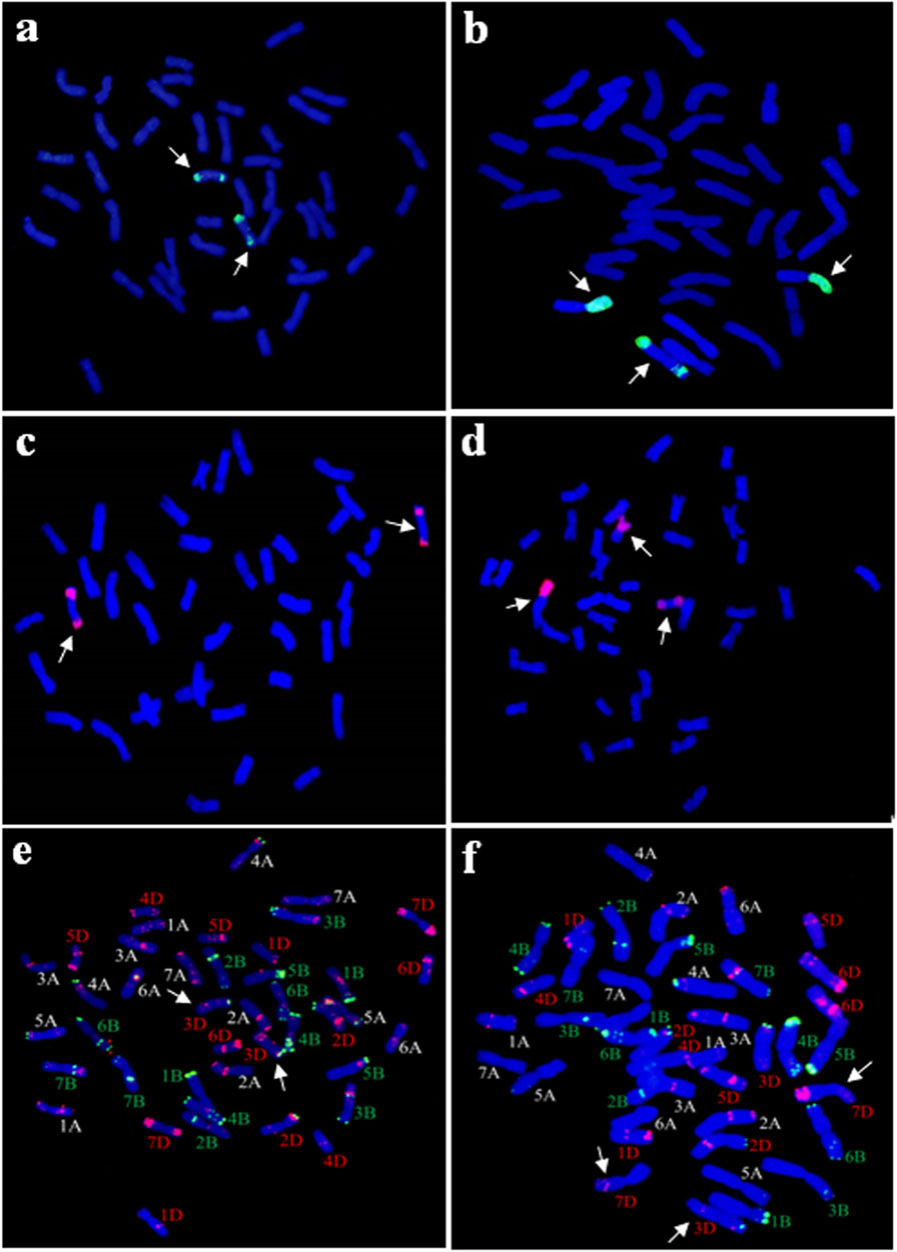
GISH (**a**–**d**) and FISH (e–f) identification of K15–1192-2 (**a**, **c**, **e**) and K15–1194-2 (**b**, **d**, **f**). The probes used for in situ hybridization were *E. repens* genomic DNA (a, b); *Pse. strigosa* genomic DNA (**c**, **d**); pSc119.2 and pTa535 (**e**, **f**). Arrows indicate the wheat–*E.repens* translocational chromosomes

P1142-1-2 simultaneously probed with *Pse. strigosa* (St genome) and *H. bogdanii* (H genome) DNA showing the complete 42 chromosomes of wheat, two St-genome chromosomes, four chromosomes with wheat, H, and St genome large fragments, plus eight wheat chromosomes with double St-genome terminal translocations (Fig. 2). When *E. repens* total genomic DNA was used as the probe and J-11 genomic DNA as the block, we observed 40 wheat chromosomes plus two wheat–*E. repens* double terminal translocational chromosomes in K15–1192-2 (Fig. 3a). K15–1194-2 (2*n* = 42) was found to have a pair of wheat–*E. repens* Robertsonian translocations and one double terminal translocational chromosome (Fig. 3b). To further determine the identities of the alien chromosomes involved in the translocations, GISH was also performed using the total genomic DNA of *H. bogdanii* and *Pse. strigosa* as the probe. However, *H. bogdanii* chromatin was not detected in the two lines. Thus, the wheat–*E. repens* translocations involved the St genome by virtue of the signals appearing on the translocated chromosomes of the two lines (Fig. 3c, d).

To further determine the identity of the wheat chromosomes involved in the translocations, FISH was performed on the translocation using pSc119.2 and pTa535 probes. We revealed that the double terminal translocated fragment of line K15–1192-2 was situated on wheat chromosome 3DS and 3DL (Fig. 3e). Line K15–1194-2 was identified as harboring a pair of 7DS/?StL Robertsonian translocations and one 3D/?St double terminal translocational chromosome (Fig. 3f).

### Molecular marker analyses

**Fig. 4:**
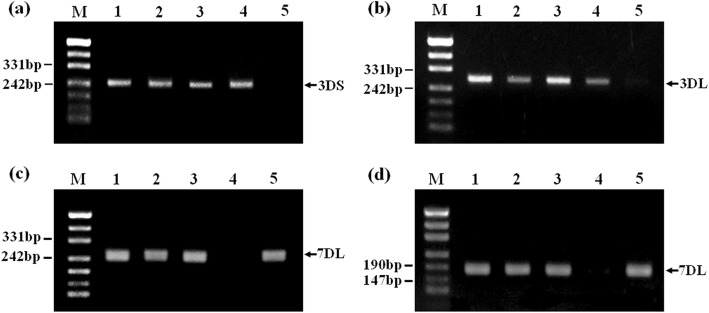
Amplification patterns of wheat SSR markers. **a**, Chromosome 3DS SSR marker *Xcfd64*; **b**, Chromosome 3DL SSR marker *Xcfd211*; **c**, Chromosome 7DL SSR marker *Xwmc14*; **d**, Chromosome 7DL SSR marker *Xbarc111*. Arrows indicate the amplification products diagnostic for wheat D -genome chromosomes. M, DNA ladder; 1, CS; 2, CN16; 3, P1142-1-2; 4, K15–1194-2; 5, K15–1192-2

The amplification products for the terminal regions of wheat chromosomes 3DS-and 3DL-SSR specific markers (i.e., *Xcfd64* and *Xcfd211*) were observed in K15–1194-2, P1142-1-2, CS, and CN16. In contrast, no amplicons were generated for K15–1192-2 by these two primers (Fig. 4a, b). The SSR analysis also indicated that amplification with two primer pairs (*Xwmc14* and *Xbarc111*), which were specific for chromosome 7DL, yielded bands of the expected size from K15–1192-2, P1142-1-2, CS, and CN16 but not from K15–1194-2 (Fig. 4c, d).

**Fig. 5:**
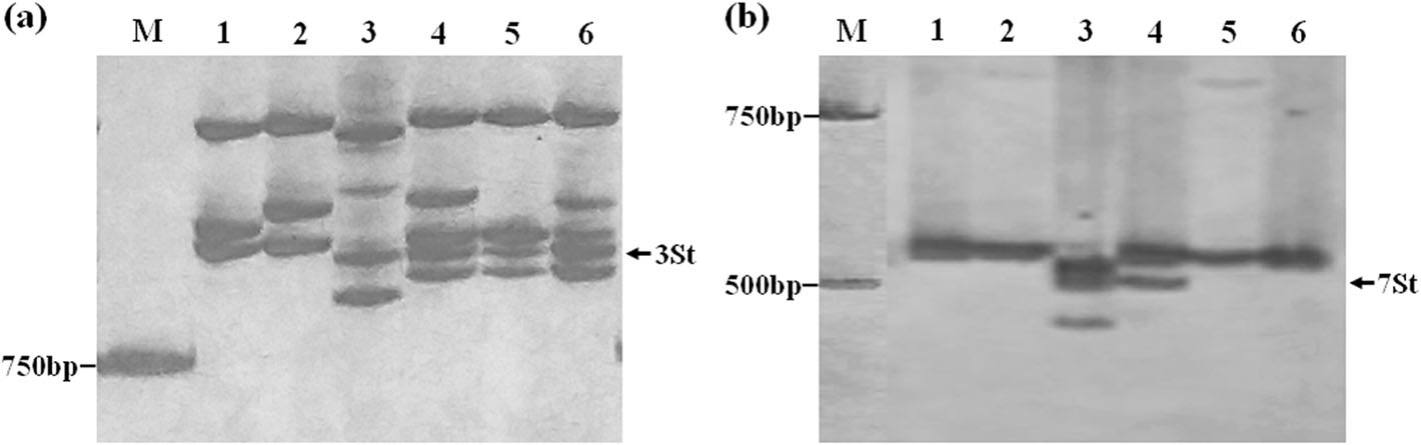
Amplification patterns of EST-SSR markers *3EST-147* (**a**) and *7EST-133*(**b**). Arrows indicate the amplification products diagnostic for *E. repens* St-genome chromosomes. M, DNA ladder; 1, CS; 2, CN16; 3, *E. repens*; 4, P1142-1-2; 5, K15–1194-2; 6, K15–1192-2

Kong et al. [22] developed the molecular markers specific to 1St–7St chromosome of *Roegneria ciliaris* according to ESTs of wheat. Using these EST-SSR markers, we showed that the marker *3EST-147* specific for 3St chromosome (ID of ESTs: BF293133) amplified common band (760 bp) in *E. repens*, P1142-1-2, K15–1192-2, and K15–1194-2, but this band was not amplified in CS and CN16 (Fig. 5a). In contrast, the specific bands amplified by the EST-SSR markers specific for chromosome 1St, 2St, 4St, 5St, 6St, and 7St were not detected between *E. repens* or P1142-1-2 and K15–1192-2, K15–1194-2 (Fig. 5b). Those results confirmed that the alien chromatin involved in both translocations was derived from 3St chromosome of *E. repens*.

### Morphology of K15–1192-2 and K15–1194-2

K15–1192-2 and K15–1194-2 displayed stable morphological traits between the parents CN16 and P1142-1-2 (Table 1). Their average plant height were significantly greater than those of CN16 or P1142-1-2. The tiller number of the two lines was significantly greater than that of CN16, and similar to that of P1142-1-2. The average spike length of K15–1194-2 were significantly greater than that of P1142-1-2, CN16, and K15–1192-2. The thousand-kernel weight of K15–1192-2 and K15–1194-2 was significantly greater than that of P1142-1-2, but lower than that of CN16. No significant differences in spikelets per spike were observed among K15–1192-2, K15–1194-2, and either CN16 or P1142-1-2.

**Table 1:** Agronomic traits of K15–1192-2 and K15–1194-2 and their parental lines

Lines	Plant height (cm)	Tiller number	Spike length (cm)	Spikelet per spike	Grains per spike	Seed setting rate (%)	Thousand-grain weight
P1142-1-2	89.7 ± 2.7b	12.3 ± 1.3a	9.7 ± 1.0b	22.5 ± 2.0a	27.7 ± 1.5b	61.6 ± 13.4c	19.4 ± 0.4d
CN16	75.2 ± 1.3c	9.4 ± 1.1b	11.3 ± 1.0ab	20.3 ± 1.2a	38.3 ± 3.5a	94.3 ± 3.6a	41.0 ± 0.5a
K15–1192-2	96.1 ± 3.8a	11.9 ± 0.9a	10.1 ± 1.5b	19.6 ± 1.7a	27.8 ± 6.4b	70.9 ± 4.7b	28.5 ± 1.7c
K15–1194-2	100.5 ± 4.6a	12.5 ± 2.2a	12.2 ± 1.4a	22.3 ± 2.0a	24.5 ± 8.6b	54.9 ± 6.2d	34.3 ± 0.9b

Data in the columns indicate means ± standard errors

Lowercase letters following the means indicate significant differences at *P* < 0.05 as determined by the least significant differences

### FHB resistance evaluation

**Fig. 6:**
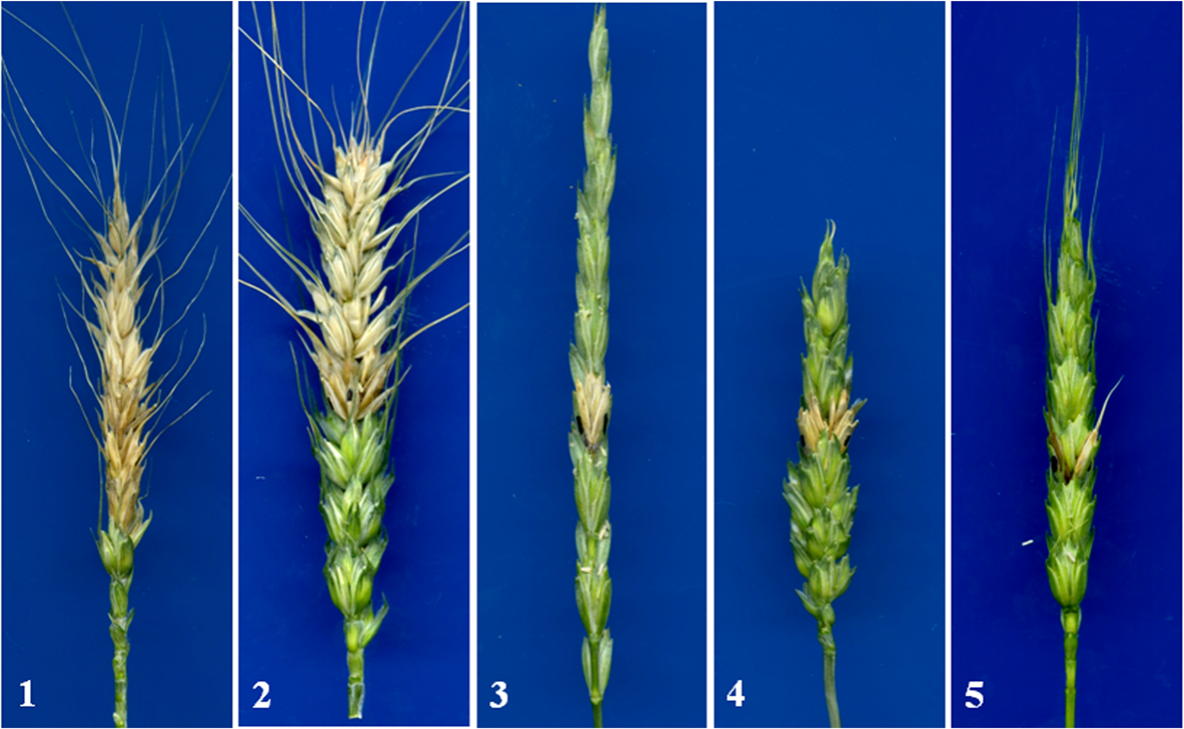
Symptoms on K15–1192-2, K15–1194-2, and the controls at 21 days after inoculation with *Fusarium graminearum* spores. 1, SY95–71; 2, CN16; 3, P1142-1-2; 4, K15–1194-2; 5, K15–1192-2

K15–1192-2, K15–1194-2, P1142-1-2, CN16, and SY95–71 were evaluated for FHB resistance in the controlled-environment room. The susceptible comparison line SY95–71 and the parents CN16 and P1142-1-2 had mean 93.3, 58.8, and 7.7% infected florets, respectively. In contrast, K15–1194-2 and K15–1192-2 were resistant (Fig. 6), with mean infection rates of 7.1 and 8.3%, respectively, similar to that of P1142-1-2.

### Stripe rust resistance

**Fig. 7:**
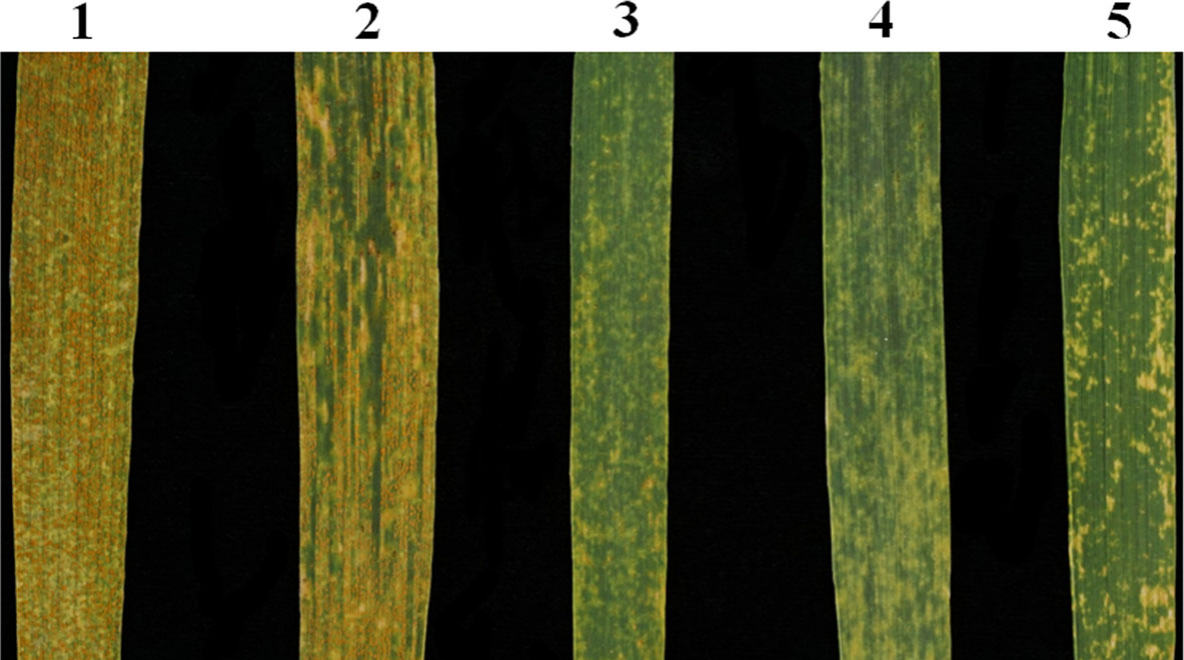
Stripe rust resistance of K15–1192-2, K15–1194-2, and the controls at adult plant stages. 1, SY95–71; 2, CN16; 3, P1142-1-2; 4, K15–1194-2; 5, K15–1192-2

**Fig. 8:**
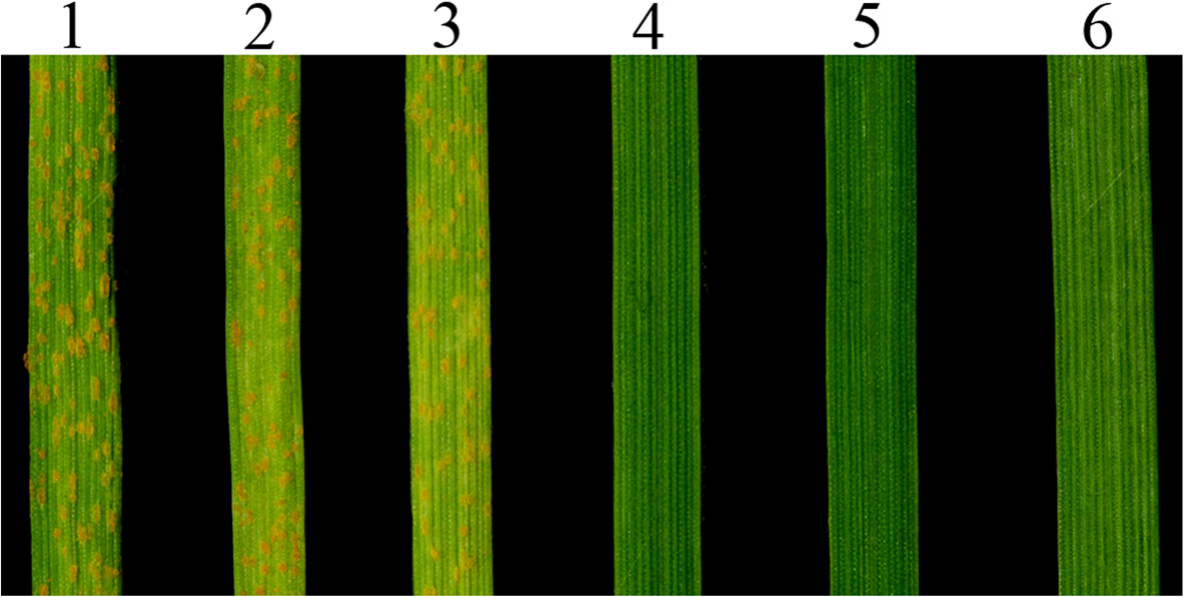
Stripe rust resistance of K15–1192-2, K15–1194-2, and the controls at seedling stages. 1, SY95–71; 2, Crocus; 3, CN16; 4, P1142-1-2; 5, K15–1194-2; 6, K15–1192-2

At the adult plant stages, each plant of the three replications of lines CN16 and SY95–71 were susceptible to a mixture of *Pst* races CYR-32, CYR-33, CYR-34, and V26/Gui22–14, both showing infection types 4. In contrast, P1142-1-2, K15–1192-2, and K15–1194-2 plants were highly resistant to these races, all showing infection types 0; (Fig. 7). The stripe rust evaluation at seedling stage indicated that P1142-1-2, K15–1192-2, and K15–1194-2 were highly resistant to *Pst* race CYR-34. In contrast, SY95–71, and the parental lines CN16 and Crocus were highly susceptible (Fig. 8).

## Discussion

Most translocational genotypes between wheat and wild related species from genera *Aegilops*, *Secale*, *Hordeum*, *Thinopyrum*, *Agropyron*, *Dasypyrum*, *Leymus*, and *Psathyrostachys* have been successfully produced in current wheat breeding programme [23–25]. *Elymus* L. sensu lato species possess resistance or tolerance to various biotic and abiotic stresses, and it serves as an important wild gene pool that can increase the genetic diversity of common wheat [26, 27]. The production of wheat–*Elymus* compensating translocations with targeted alien chromosomes is the first important step for transferring genes from *Elymus* species to wheat for breeding purposes. To date, some introgressions from *Elymus* species into wheat have been produced, including *E. trachaycaulus*, *E. ciliaris*, and *E. tsukushiense* [9, 26–28]. Wide crosses between *E. repens* and common wheat began in the 1980s [29]. Zeng et al. [21] developed and characterized eight common wheat*–E. repens* BC_1_F_9_ progeny lines, and numerous translocational chromosomes were detected in these lines. Among these lines, P1142-1-2 (2*n* = 56) contains 42 wheat chromosomes and 14 translocational chromosomes invovling the W-St-H and St-H translocations, which is regarded as significant breeding potential material [21]. We confimed that P1142-1-2 consisted of the complete 42 chromosomes of wheat, two St-genome chromosomes, four chromosomes with wheat, H, and St genome large fragments, plus eight wheat chromosomes with double St-genome terminal translocations. To transfer desirable traits from *E. repens* into wheat cultivars of the Sichuan Basin, China, we crossed P1142-1-2 with CN16, and the F_1_ was backcrossed with CN16. The homozygous translocation lines K15–1192-2 and K15–1194-2 were identified from the P1142-1-2/2*CN16 BC_1_F_3_ progeny lines. GISH and FISH analyses of somatic metaphase chromosomes confirmed the presence of a wheat–*E. repens* 3D/?St double terminal translocation and 7DS/?StL Robertsonian translocation, respectively. Fedak et al. [30] indicated that P1142-3-15 also had 42 chromosomes with one pair of chromosomes showing terminal translocations on both 3D arms. However, it was obtained from the common wheat Crocus/*E. repens*//Crocus F_9_ progeny lines. Furthermore, The line K15–1192-2 had an average of 20.57 bivalents and 0.86 univalents, but the lower incidence of univalents (0.34) were observed in line P1142-3-15 [21]. The FHB resistance of K15–1192-2 had infection rates of 8.3% compared with P1142-3-15 at 11.46% [21]. Therefore, K15–1192-2 and P1142-3-15 are the different translocation lines though they have the terminal translocations on both 3D arms. We further confirmed that the wheat–*E. repens* translocations in K15–1192-2 and K15–1194-2 involved the 3St chromatin using St–specific EST-SSR markers, which was consistent with the amplification results of Kong et al. [22]. These results, together with the high cytological stability of the two lines, indicated that the chromatin that was transferred from 3St of *E. repens* compensated for the lack of wheat chromatin. These lines provide appropriate bridge–breeding–materials for alien gene introgression to improve wheat disease resistance.

Resistance to FHB has been a major focus of wheat breeding efforts for many decades and has relied on diverse germplasm resources [31]. Sources of FHB resistance used in current wheat breeding programs can be traced to limited parents, including Sumai 3 and its derivatives, Wangshuibai and Wuhan 1 [6]. Therefore, there is a constant need for evaluating and identifying new sources of resistance in alien germplasm as well as in wheat [9]. Liu et al. [32] indicated that *Roegneria ciliaris*, *Roegneria kamoji*, and *L. racemosus* had high levels of resistance to FHB. Fu et al. [33] found a FHB resistance gene located on chromosome 7E that was derived from *Th. elongatum*, but it was not used owing to linkage drag. *Fhb3* was derived from a tetraploid wheat relative, *L. racemosus*, and was transferred to wheat in the form of a compensating Robertsonian translocation T7AL.7Lr#1S [8]. Cainong et al. [9] successfully mapped and transferred the *Fhb6* from *E. tsukushiensis* into wheat. Guo et al. [10] developed and characterized secondary 7DS.7el_2_L translocation lines with shortened *Th. ponticum* segments carrying *Fhb7*. FHB resistance has also been documented in *Aegilops tauschii*, *E. trachaycaulus*, *E. repens*, *R. ciliaris*, *Th. junceum*, *Triticum monococcum*, *Triticum timopheevii*, *Triticum carthlicum*, and *Triticum macha* [22, 27, 30, 31, 34]. Recently, Fedak et al. [30] revealed that FHB resistance was provided by the wheat–*E. repens* translocation on the long arm of chromosome 3D. Zeng et al. [21] reported that eight wheat–*E. repens* introgression lines expressed high levels of resistance to FHB, and line P1142-1-2 (2*n* = 56) displayed an 11.35% infection rate. At present, most wheat cultivars and breeding lines from the Sichuan Basin, China are susceptible to FHB. To provide novel FHB resistance resources and transfer new genes to wheat cultivars grown in Sichuan, we developed and characterized two new wheat–*E. repens* homozygous translocation lines (2*n* = 42) from P1142-1-2/2*CN16 generation. Compared with the infection rate of the parent CN16 (58.8%), K15–1192-2 (8.3%) and K15–1194-2 (7.1%) were highly resistant to FHB. Therefore, the new translocation lines may represent valuable germplasm for breeding FHB-resistant wheat cultivars.

New stripe rust races CYR34 is effective against all the previously identified *Pst* races and has been deployed in commercial cultivars to fight predominant races of the fungus in China [15]. Very few genes are effective against CYR34, including *Yr5*, *− 15*, *− 16*, *− 18*, and the original sources of *Yr11*, *− 12*, *− 13*, and *− 14* [35]. This pathogen represents a serious threat to wheat production in the Sichuan Basin of China and potentially in other regions [15]. It is necessary to accelerate progress in identifying and utilizing new effective stripe rust resistance genes to develop new wheat varieties with durable resistance [16]. The wild relatives of wheat provided abundant and diverse resistance resources, such as *Yr15*, *− 24/26*, *− 35*, *− 36*, *− 53*, *− 64*, and *− 65* from tetraploid wheat, *Yr9* from *S. cereale*, *Yr8*, *− 17*, *− 28*, *− 37*, *− 38*, *− 40*, *− 42*, *− 48*, and *− 70* from *Aegilops* species, and *Yr50* from *Th. intermedium* [18]. In this study, K15–1192-2 and K15–1194-2, which were derived from P1142-1-2/2*CN16 BC_1_F_3_ progenies, were highly resistant to prevalent Chinese *Pst* races at seedling and adult plant stages. The parent P1142-1-2 is resistant to all the tested races, while CN16 is susceptible. P1142-1-2 was derived from the cross ‘Crocus’/*E. repens*//‘Crocus’ [21]. A survey of stripe rust resistance revealed that ‘Crocus’ was susceptible, and the resistance of P1142-1-2 was derived from *E. repens*. Therefore, this pedigree provides the only evidence that K15–1192-2 and K15–1194-2 carry the stripe rust resistance gene from 3St chromosome of *E. repens*. As far as we know, this is the first demonstration of a successful transfer of a new and high-level stripe rust resistance gene from *E. repens* that involves the St genome. The new wheat lines offer a novel resource for improving resistance to all the prevalent races of stripe rust present in the Sichuan Basin of China.

## Conclusions

In conclusion, we developped and characterized two wheat*–E. repens* 3St chromosomal translocation lines by GISH, FISH, SSR, and EST-SSR markers. Compared with the wheat parent, these translocation lines expressed high levels of resistance to FHB and stripe rust pathogens prevalent in China. Our study has determined that the chromosome 3St of *E. repens* harbors gene(s) highly resistant to FHB and stripe rust, and chromatin of 3St introgressed into wheat chromosomes completely presented the resistance, indicating the feasibility of using these translocation lines as novel material for breeding resistant wheat cultivars and alien gene mining.

## Methods

### Plant materials

**Fig. 9:**
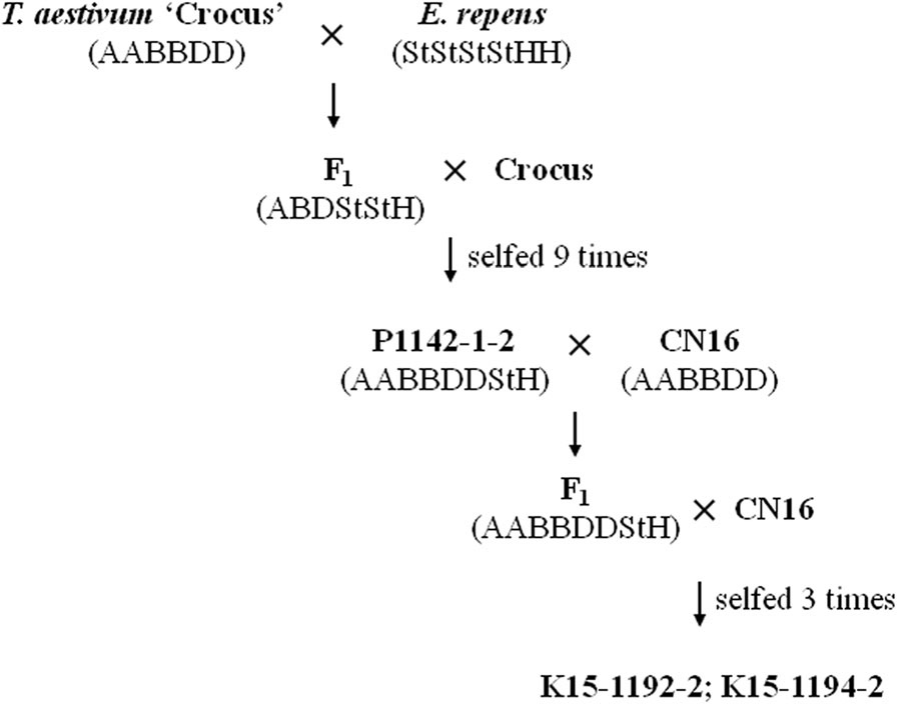
Pedigree details of K15–1192-2 and K15–1194-2

The wheat–*E. repens* line P1142-1-2 (2*n* = 8*x* = 56), which has the characteristics of tolerance to cold, a variety of soil types, and heavy metals, as well as superior resistance to FHB and rust, was originally developed and identified from the crosses Crocus/*E. repens*//Crocus BC_1_F_9_ progenies at the Eastern Cereal and Oilseed Research Center, Ottawa, Canada [21]. The native wheat cultivar Chuannong16 (CN16) is ideal recurrent parents for wheat breeding programs in southwestern China because it possesses a comprehensive array of good agronomical characteristics; however, it is susceptible to stripe rust, powdery mildew, and FHB [24]. To produce wheat–*E. repens* derivative line, we crossed P1142-1-2 with CN16, and the F_1_ was backcrossed with CN16. Then seeds selected from the BC_1_F_1_ plants were bulked and advanced to the BC_1_F_3_ generation by single seed descent. Two derivative lines K15–1192-2 and K15–1194-2, with high resistance to stripe rust and FHB over 2 years of observation, were isolated from the 76 P1142-1-2/2*CN16 BC_1_F_3_ progeny lines (Fig. 9). Wheat line SY95–71 and Crocus was used as a susceptible control for diaease response tests. Wheat cultivar Chinese Spring (CS) was used as a positive control for the molecular marker analysis. Wheat cultivar J-11 was used as a source of blocking DNA, and *E. repens* accession PI229925, *Hordeum bogdanii* (2*n* = 2*x* = 14, HH) accession Y1819, and *Pseudoroegneria strigosa* accession W6–14049 (2*n* = 2*x* = 14, StSt) were used as sources of probes DNA in GISH analysis. The voucher specimens have been deposited at herbarium of Triticeae Research Institute, Sichuan Agricultural University, China (SAUTI).

### Meiosis analysis

The analysis of meiosis followed the procedures described by Kang et al. [36]. At least 50 pollen mother cells (PMCs) were observed for each plant. The images were captured with a DP-70 CCD camera using Olympus BX-63 microscope (Olympus, Tokyo, Japan).

### GISH and FISH analysis

Root tips were treated and digested with the procedure of Komuro et al. [37], then the slides were prepared for GISH as described by Han et al. [38]. Genomic DNAs were extracted from fresh leaves of *E. repens*, *H. bogdanii*, *Pse. strigosa*, and wheat cultivar J-11 by the cetyltrimethylammonium bromide method [39]. *E. repens*, *H. bogdanii*, and *Pse. strigosa* DNAs were independently labeled with both fluorescein-12-dUTP (green) and Texas Red-12-dUTP (red) by the nick translation method (Thermo Fisher Scientific, Eugene, OR, USA), then used as the hybridization probes. GISH analysis was performed according to Han et al. [40] with a probe DNA to blocking agent DNA ratio of 1:150. Ten μL of hybridization solution containing 2× SSC (saline sodium citrate), 10% dextran sulphate, 10 ng/μL of labeled probe DNA together with blocking DNA were loaded per slide, denatured by heating at 85 °C for 5 min, incubated for 8 h at 37 °C, and washed in 2× SSC at room temperature. Finally, the chromosomes were counterstained with 4,6-diamino-2-phenylindole solution (Vector Laboratories, Burlingame, CA, USA). The GISH images were captured with a DP-70 CCD camera using Olympus BX-63 microscope.

The GISH slides were washed with 70% (v/v) ethanol for 5 min, 2× SSC at 60 °C for 30 min, ddH_2_O (double distilled water) for 10 min, and 100% (v/v) ethanol for 5 min, respectively. FISH analysis was subsequently used to identify the constitution of the lines K15–1192-2 and K15–1194-2, using pSc119.2 and pTa535 as probes [37, 41]. FISH was conducted according to Han et al. [38]. FISH signals were visualized under a fluorescence microscope (Olympus BX63), and images were captured by DP-70 CCD camera and analyzed by Adobe Photoshop software.

### Molecular marker analysis

The specific Simple sequence repeat (SSR) primer pairs of the wheat D-genome chromosomes [42] were used to determine the wheat chromatin of K15–1192-2 and K15–1194-2. The expressed sequence tag-SSR (EST-SSR) primer pairs distributed in the seven homoeologous groups of wheat [22] were used to identify the alien chromatin in both lines. All the PCR primers were synthesized by TSINGKE (Chengdu, China), and the details are shown in Table 2. CS, P1142-1-2, and CN16 were used as controls. The PCR amplification were conducted as previously described [22, 42].

**Table 2:** Sequences of wheat SSR and EST-SSR markers

Marker	Primer sequence (5′-3′)	Annealing temperature (°C)	Arm location	ID of ESTs	References
*Xwmc14*	F: ACCCGTCACCGGTTTATGGATG R: TCCACTTCAAGATGGAGGGCAG	63	7DL(terminal)	–	Somers et al. 2004
*Xbarc111*	F: GCGGTCACCAGTAGTTCAACA R: GCGTATCCCATTGCTCTTCTTCACTAAC	60	7DL (proximal)	–	Somers et al. 2004
*Xcfd211*	F: AGAAGACTGCACGCAAGGAT R: TGCACTAAAGCATCTTCGTGTT	65	3DL (terminal)	–	Guyomarc’h et al. 2002
*Xcfd64*	F: ACAGTGTTGTTGCCCCTTTC R: CCCATGTTACAGCTTTGGGT	64	3DS (terminal)	–	Guyomarc’h et al. 2002
*1EST-255*	F: CCAGGACAGCCTATCCAAGA R: TCGAAGTTGGACTTCAGCAA	57	1AL 1BL 1DL	Ta#S16058339	Kong et al. 2018
*1EST-1134*	F: CACAAACTATCCAAAGGATGA R: GTGGAACATTTTCAGGTGAC	55	1AS 1DS	BG605065	Kong et al. 2018
*2EST-983*	F: ACAGGAGGTTGGATGAGTGG R: TCCACGTGTGTTTCGTCAAT	57	2AS 2BS 2DS	BG314234	Kong et al. 2018
*2EST-705*	F: AGGTCACTGCAGGAGGAGGA R: GAAAAGATGATGAGCTGGTCTGG	55	2BL 2DL	BF293175	Kong et al. 2018
*3EST-186*	F: CAATTTGTTGCCTACGTCA R: AGTTCTAATGGTGACCCACA	55	3AL 3BL 3DL	BE406551	Kong et al. 2018
*3EST-147*	F: AAGCTCGTCTTCATCGTCTA R: GTACAGCCCCAGCAGGTA	55	3AS 3BS 3DS	BF293133	Kong et al. 2018
*4EST-100*	F: GTGCACTCCGTCGAAGCTA R: AGGAGCTGGTGATGAACTGG	58	4AS 4BL 4DL	BE497134	Kong et al. 2018
*4EST-19*	F: GTACGTAGCAGCCGATGGAT R: CCCCGATCGAGAAGTTACAA	57	4AL 4DL	BE637934	Kong et al. 2018
*5EST-79*	F: AAGTATGCAGCCAGATCTCA R: GGTTATTGCTCTTGCAGATG	54	5AL 5BL 5DL	BQ280540	Kong et al. 2018
*5EST-10*	F: GAGCTGGATCTTCAGCCTAT R: AATTTTTGCCATGAGATCG	53	5AL	BM137728	Kong et al. 2018
*6EST-358*	F: GTACCATTCGATTGTTCTGC R: GGAAATCCTATGCCCTTAAT	55	6AL 6BL 6DL	BE399146	Kong et al. 2018
*CINAU15*	F: AGATCCAACACCAGTTCAAG R: ATGTTATGGAGGCTTGTGTC	53	6AS 6BS 6DS	Contig17515	Kong et al. 2018
*7EST-133*	F: CTCTTCCCCTCTCTCGTCCT R: GCTCCAAATCTTCACCAAGC	57	7DS	Ta#S13057851	Kong et al. 2018
*7EST-138*	F: GATTAGGCAAATGGGTCA R: CTCATCGGGTTCAGTGGT	55	7AS 7BS 7DS	BE494425	Kong et al. 2018

*F* forward primer; *R* reverse primer

### Agronomical traits evaluation

The seven morphological traits (including plant height, tiller number, spike length, spikelet per spike, grains per spike, thousand-grain weight, and seed setting rate) of K15–1192-2 and K15–1194-2 and their parents were evaluated in a field trial in Wenjiang, Sichuan Province, China with three replications. The detailed method was performed as described by Kang et al. [24]. Significant differences in traits were determined using the SAS 8.2 system (SAS Institute Inc., Cary, NC, USA).

### Evaluation of FHB resistance

The plant inoculation experiments were performed according to Qi et al. [43]. At the mid-anthesis stage, two florets of a single central spikelet were point-inoculated with a 10-μL solution with one thousand *F. graminearum* conidia in distilled water. The inoculated spikes were sprayed with water and wrapped in plastic film for 48 h to maintain humidity. The wheat plants were incubated at 25 °C in a controlled-environment room. Head blight symptoms were assessed at 21 days after inoculation, with 5–10 plants per treatment.

### Stripe rust resistance screening

K15–1192-2, K15–1194-2, P1142-1-2, CN16, and SY95–71 were evaluated for adult plant responses to a mixture of *Pst* races (CYR-32, CYR-33, CYR-34, and V26/Gui22–14) in a field trial using the smear method [44] in the growing season 2017–2018 at Chengdu, Sichuan, China. The evaluation was conducted according to Kang et al. [24] with three replications. For each replication, 20 grains of each line were evenly planted in 2.0 m rows, spaced 0.3 m apart. K15–1192-2, K15–1194-2, P1142-1-2, CN16, Crocus, and SY95–71 were evaluated for seedling stage reacctions to *Pst* race (CYR-34) under growth chamber. The plants were inoculated and evaluated as described by Li et al. [45]. Wheat line SY95–71 was used as susceptible control. Stripe rust infection type (IT) was identified as described by Li et al. [45].

## Data Availability

The datasets generated and analyzed during the present study are available from the corresponding author on reasonable request.

## Abbreviations

CN16: Chuannong16
EST-SSR: Expressed sequence tag–simple sequence repeat
FHB: Fusarium head blight
FISH: Fluorescence in situ hybridization
GISH: Genomic in situ hybridization
PCR: Polymerase chain reaction
PMCs: Pollen mother cells
*Pst*: *Puccinia striiformis* f. sp. *tritici*
SSRs: Simple sequence repeats
